# Dissemination of bloodmeal acquired *Rickettsia felis* in cat fleas, *Ctenocephalides felis*

**DOI:** 10.1186/1756-3305-6-149

**Published:** 2013-05-24

**Authors:** Chutima Thepparit, Supanee Hirunkanokpun, Vsevolod L Popov, Lane D Foil, Kevin R Macaluso

**Affiliations:** 1 Department of Pathobiological Sciences, Louisiana State University, School of Veterinary Medicine, Baton Rouge, LA 70803, USA; 2 Center for Vaccine Development, Institute of Molecular Biosciences, Mahidol University, Nakhon Pathom 73170, Thailand; 3 Department of Pathology, Center for Biodefense and Emerging Infectious Diseases, Institute for Human Infections and Immunity, The University of Texas Medical Branch, Galveston, TX 77555, USA; 4 Department of Entomology, Louisiana State University, Baton Rouge, LA 70803, USA

**Keywords:** *Rickettsia felis*, *Ctenocephalides felis*, Cat flea, Rickettsial infection

## Abstract

**Background:**

Cat fleas, *Ctenocephalides felis*, are known biological vectors for *Rickettsia felis*. Rickettsial transmission can be vertical via transovarial transmission within a flea population, as well as horizontal between fleas through a bloodmeal. The previously undescribed infection kinetics of bloodmeal-acquired *R. felis* in cat fleas provides insight into the *R. felis*-flea interaction.

**Findings:**

In the present study, dissemination of *R. felis* in previously uninfected cat fleas fed an *R. felis*-infected bloodmeal was investigated. At weekly intervals for 28 days, rickettsial propagation, accumulation, and dissemination in gut epithelial cells, specifically in the hindgut and the specialized cells in the neck region of midgut, were observed on paraffin sections of infected cat fleas by immunofluorescence assay (IFA) and confirmed by PCR detection of *R. felis* 17-kDa antigen gene. IFA results demonstrate ingested rickettsiae in vacuoles during early infection of the gut; lysosomal activity, indicated by lysosome marker staining of freshly-dissected gut, suggests the presence of phagolysosome-associated vacuoles. Subsequent to infection in the gut, rickettsiae spread to the hemocoel and other tissues including reproductive organs. Densely-packed rickettsiae forming mycetome-like structures were observed in the abdomen of infected male cat fleas during late infection. Ultrastructural analysis by transmission electron microscopy (TEM) confirmed the presence and infection characteristics of *Rickettsia* including rickettsial destruction in the phagolysosome, rickettsial division, and accumulation in the flea gut.

**Conclusions:**

This study intimately profiles *R. felis* dissemination in cat fleas and further illuminates the mechanisms of rickettsial transmission in nature.

## Findings

### Background

*Rickettsia felis* has emerged as a cosmopolitan arthropod-borne pathogen infecting various arthropod hosts. First associated with human disease in 1991, this intracellular Gram negative bacteria shares clinical signs with other rickettsial diseases, ranging from fever and generalized maculopapular rash to more severe acute polyneuropathy-like symptoms [1,2]. Coinciding with the impetus to understand transmission biology, increased recognition of *R. felis* as a pathogen derives from studies revealing *R. felis* in approximately 6% of non-malarial, febrile Senegalese and Kenyan patients hospitalized [3,4].

Rickettsial transmission by arthropod vectors can be vertical, horizontal, or for dynamic organisms such as *R. felis*, both. In an endosymbiont relationship, some arthropod hosts and their respective *Rickettsia* spp. are strictly maintained vertically between life cycle stages typically providing a benefit to the host [5]. Alternatively, *Rickettsia* spp. conferring a negative fitness effect require horizontal transmission for maintenance in arthropod populations. Identification of both transmission paradigms is unique to *R. felis*. For example, in non-hematophagous insect hosts, *R. felis* infection is associated with parthenogenesis and maintained 100% transovarially. Clearance of the organism from adult *Liposcelis*, common booklice, results in decreased longevity, fecundity, and non-viable egg production [6,7]. Conversely, in the cat flea, *Ctenocephalides felis*, vertical transmission of *R. felis* varies, not influencing flea fitness, and horizontal transmission via cofeeding is essential for rickettsial maintenance in flea populations (reviewed in [8]).

While molecular methods confirm detection of *R. felis* in a variety of hematophagous arthropods, only cat fleas are known biological vectors. A disseminated infection exists in vertically infected fleas; *R. felis* has been identified by PCR and microscopy in the midgut epithelial cells (adult and larval fleas), as well as adult flea muscle cells, fat body, tracheal matrix, ovaries, epithelial sheath of testes, and salivary glands [9-11]. Despite the known transmission potential of cat fleas for *R. felis*, the infection kinetics of horizontally-acquired *R. felis* in cat fleas remains unexamined. The ability to infect cat fleas with *R. felis* via an infectious bloodmeal [12] and show transmission of *R. felis* between fleas [13] was recently demonstrated using an artificial host system. To better understand the infection process, the objective of the current study involved monitoring rickettsial infection in naïve fleas as it was acquired via an infectious bloodmeal. A combination of PCR, immunofluorescence assay (IFA), and transmission electron microscopy (TEM) techniques were employed to track novel infection over the duration of the cat flea’s adult life. Through mapping the infection process in the natural host, an appreciation of the ecology of this emerging pathogen and potential points of intervention may be identified.

### Methods

#### Source of fleas and rickettsial cultivation

Cat fleas (*C. felis* Bouche) purchased from Elward II (EL-Labs, Soquel, CA) were reared as previously described [12]. The Louisiana State University (LSU) strain of *R. felis* was propagated in ISE6, *Ixodes scapularis*-derived cells, and the *R. felis*-infected bloodmeal preparation was carried out as described [13] prior to enumeration using *Bac*Light viability stain kit (Molecular Probes). A negative bloodmeal control was prepared from uninfected ISE6 cells in the same manner.

#### Experimental design

Newly emerged, sex-separated cat fleas were pre-fed with heat-inactivated, defibrinated bovine blood for 24 hr prior to exposure to the *R. felis*-infected or uninfected bloodmeal at a dilution of 8.6 × 10^10^ rickettsiae per ml. After 24 hr exposure, female and male fleas in each experimental group were mixed equally and maintained on defibrinated bovine blood (non heat-inactivated) with fresh blood replaced as needed for the duration of the experiment. Fleas, for the rickettsial dissemination examination, were collected at weekly intervals for 28 days after mixing the population [designated as day post-exposure (dpe)].

#### Genomic DNA isolation and PCR amplification

Whole fleas or individual tissues including female gut, ovary, salivary glands, and male rectal ampulla from three fleas were pooled and gDNA was extracted from the ground tissue using the DNeasy blood & tissue kit (Qiagen) according to the manufacturer’s protocol. PCR conditions for detection of the rickettsial 17-kDa antigen gene were described previously [14].

#### Microscopy

Flea tissues (described above) were dissected from three fleas and placed in fixative; TEM was carried out at University of Texas Medical Branch (UTMB) as described previously [15].

#### Immunofluorescence assay

Collected fleas were placed in embedding cassettes and fixed for 24 hr in 10% normal formalin. Formalin-fixed specimens were processed and sectioned as previously described [15]. When indicated, flea tissues were dissected and fixed with cold acetone. Rickettsiae in the formalin-fixed sections and dissected fleas tissues were detected by IFA as previously described [15].

### Results and discussion

**Figure 1:**
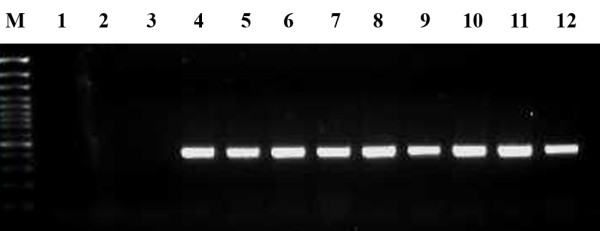
**PCR detection of rickettsial 17-kDa antigen gene in 5 dpe *R. felis*-infected, bloodmeal-acquired cat fleas.** M, 100 bp DNA marker; Lane 1, environmental blank; Lanes 2 & 3, female and male flea, respectively, fed on an uninfected blood meal; Lanes 4-7, female fleas fed on rickettsial infected blood meal; Lane 8-11, male fleas fed on rickettsial infected blood meal; Lane 12, positive PCR *R. felis* gDNA.

In order to examine the incidence of rickettsial infection of cat fleas fed on *R. felis*-infected bloodmeal, eight fleas (four male and four female) collected at 5 dpe were analyzed for the presence of rickettsial 17-kDa antigen gene by PCR. Results indicated all cat fleas exposed to an *R. felis*-infected bloodmeal were rickettsial infected (Figure 1). The absence of the 17-kDa antigen gene from either the environmental blank or the fleas fed on an uninfected bloodmeal confirmed the absence of *Rickettsia* in EL fleas, as reported previously [12,14]. The success of utilizing an artificial feeding system for persistent infection of *R. felis* on cat fleas was recently demonstrated by Reif *et al.*[12].

**Figure 2:**
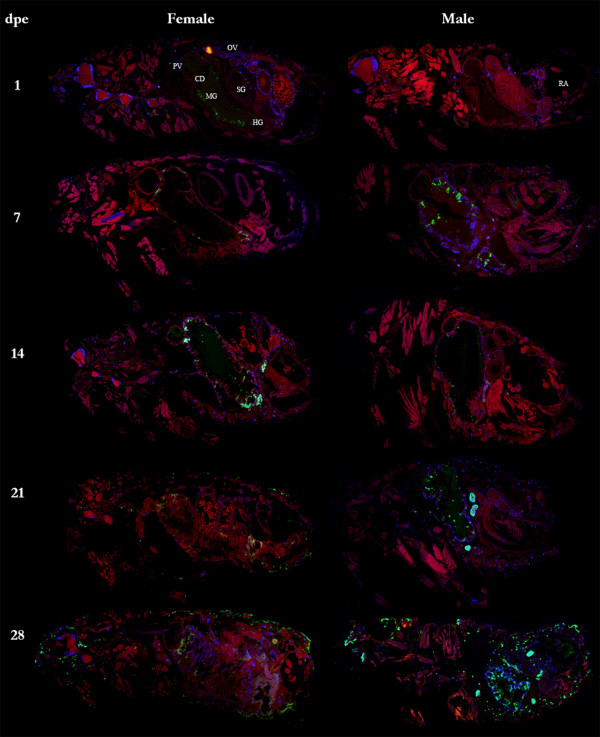
**Rickettsial dissemination in *R. felis*-infected fleas over 28 days.** 1 dpe: Ingested rickettsiae in gut and vacuoles. 7 dpe: rickettsiae occupied gut epithelium starting at the specialized cells forming the cardia (foregut/midgut junction) and the hindgut. 14 dpe: rickettsial propagation and accumulation in gut epithelium resulting in enlargement. Rickettsiae could be observed in lumen, rectal ampulla, and abdomen in a mycetome-like formation. 21-28 dpe: rickettsiae dispersed throughout body cavity and residing in other organs. CD: cardia, HG: hindgut, MG: midgut, PV: proventriculus, OV: ovaries, SG: salivary glands.

**Figure 3:**
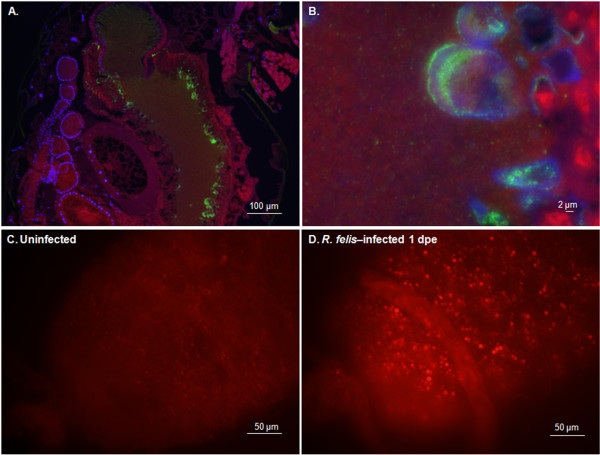
***Rickettsia *associated with phagolysosome vacuoles. A**, **B**: Rickettsiae associated with vacuoles and freely scattered rickettsiae in gut lumen during early 1 dpe. **C**, **D**: Staining of freshly dissected guts with lysosome marker (LysoTracker™) shows lysosomal activity in *R. felis*-infected flea gut suggesting the presence of phagolysosome.

**Figure 4:**
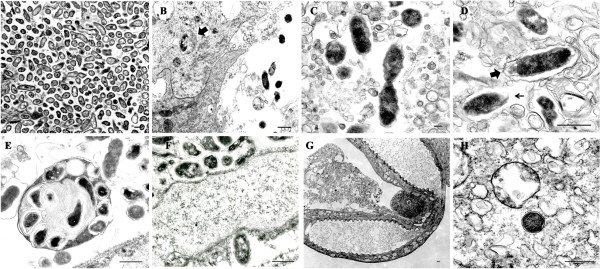
**Electron micrograph of dissected tissues of 28 days post exposure *R. felis*-infected fleas. A-D**:Gut; **A**: massive amount of rickettsiae; **B**: destroyed rickettsiae in a phagolysosome (arrow); **C**: dividing rickettsiae; **D**: rickettsiae in a vacuole (solid arrow) or escaping a vacuole (long arrow); **E-F**: rickettsiae in ovaries, **G-H**: salivary glands; **H**: typical rickettsial cell wall structure containing trilaminar membrane (cross-section). Bar=500 nm.

**Figure 5:**
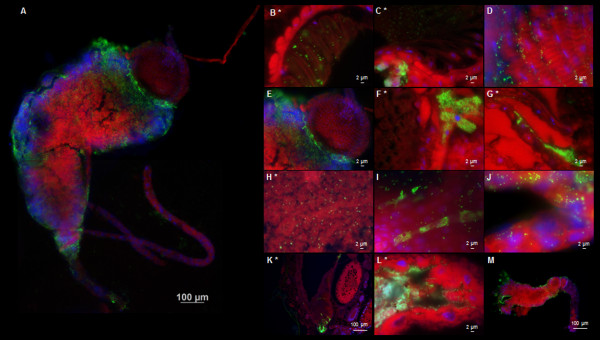
**Dissemination of *R. felis *in flea gut (7-14 days post-exposure). A**: whole dissected gut; **B-D**: proventriculus; **E-G**: specialized cells forming the cardia (foregut/midgut junction); **H**: gut lumen; **I**: connective tissues on midgut (non-permeabilized-dissected gut); **J**: Malpighian tubules; **K-M**: hindgut. * IFA on paraffin-embedded sections, otherwise on dissected tissues.

**Figure 6:**
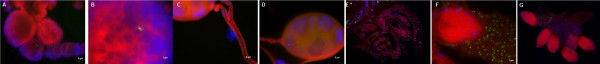
**Dissemination of *Rickettsia *in flea tissues (7-14 days post**-**exposure). A-B**: ovaries, **C-D**: salivary glands, **E-G**: rectal ampulla. * IFA on paraffin-embedded sections, otherwise on dissected tissues.

Dissemination of *R. felis* in the infected cat fleas was visualized by IFA on the paraffin-embedded cat flea sections revealing that *R. felis* spread uniformly in both female and male cat fleas over 28 dpe (Figure 2). During early *R. felis* bloodmeal acquisition, rickettsiae densely accumulated in vacuoles in flea gut lumen (Figure 2, 1 dpe; Figure 3A, B). The strong signal of lysosome marker in freshly dissected *R. felis*-infected flea gut indicated that rickettsiae were associated with the phagolysosome (Figure 3C, D); the appearance of rickettsial lysis in a vacuole was confirmed by TEM analysis. Similar to findings in the cat flea salivary glands [10], irregular-shaped rickettsiae located in double, membrane-bound vacuoles, characteristic of rickettsiae being destroyed in a phagolysosome, were observed in *R. felis*-infected flea guts and ovaries at 28 dpe (Figure 4B, E). Ultrastructural evidence of *Rickettsia*-phagolysosomal lysis was previously reported in other *Rickettsia*-host relationships [16,17]. After degradation of excessive rickettsiae, remaining rickettsiae entered gut epithelium propagating primarily in the hindgut and the specialized cells forming the cardia of midgut (foregut/midgut junction) by 7 dpe (Figure 2). Within 7-14 dpe, rickettsiae spread to the entire midgut lining after which they were detected in gut lumen and occasionally found in hindgut adnexa. Between 14-21 dpe, rickettsiae migrated to the body cavity and other organs including the excretory system. The presence of *Rickettsia* in specific organs including gut, proventriculus, salivary glands, ovaries, and rectal ampulla was confirmed by TEM (Figure 4), IFA on dissected tissue (Figures 5 and 6), and PCR detection of the rickettsial 17-kDa antigen gene (data not shown). Rickettsiae were densely aggregated around the proventriculus and its spines (Figure 5A-D). The invasion of proventriculus and midgut infection during initial infection stage was documented in tsetse fly exposed to trypanosomes via blood meal [18]. In addition to serving as a physical gut barrier, the proventriculus is thought to play a crucial role in invertebrate immunity through the expression of immune effectors and communication with other tissues to activate an adequate host immune response [19]. The evidence of clumping rickettsiae in the proventriculus and successful transmission between cat fleas suggests that *Rickettsia* may overcome the arthropod immune response [13].

**Figure 7:**
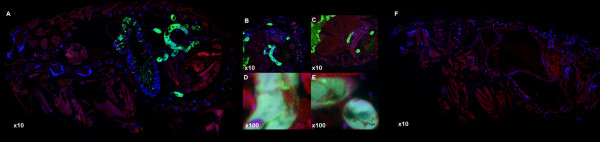
**Rickettsiae forming mycetome-like organ located in abdomen of 28 days post**-**exposure *R. felis-*infected male cat fleas. A**-**E**: *R. felis*-infected fleas, **F**: *R. felis*-uninfected flea control.

Rickettsiae disseminated among the excretory organs including hindgut, Malpighian tubules, and rectal ampulla (Figure 1; Figure 5J-M; and Figure 6F-G) supporting rickettsiae excretion in feces, a primary mechanism for horizontal transmission [13,20]. Scantly detectable, rickettsiae in cat flea ovaries (Figure 4E-F; Figure 6A-B) corresponded to the variation of transovarial transmission efficiency of *Rickettsia*. [8,21]. The visualization of rickettsiae in salivary glands confirms molecular detection of *R. felis* in these organs which may be critical for horizontal transmission [10,13]. Interestingly, after 21 dpe, dense and smaller-shaped rickettsiae formed elongated clusters similar to *Rickettsia*-associated mycetomes in booklice [15,22]. The mycetome is primarily known to be associated with nutritional symbioses supplying essential nutrition and detoxification functions to arthropod hosts [23]. The development of single-cell mycetocytes into organ-forming mycetomes in conjunction with arthropod growth stages has been described in human head and body lice. This process is associated with the transovarial transmission of endosymbionts to the offspring [24] suggestive of the possible strategy of mycetome formation in balancing symbiont populations [25]. *Rickettsia*-associated mycetome-like organs previously identified in arthropods are shown to be closely associated with primary symbionts. Mycetomic rickettsiae are thought to be transitioning from pathogen and secondary symbionts to obligate reproductive or nutritional parasites and primary symbionts [22,26,27]. Although mycetomic bacteria are associated with transovarial transmission of endosymbionts, no distinct rickettsial mycetomes in female cat fleas were reported. The restriction of mycetomic rickettsiae in the *R. felis*-infected male cat fleas (Figure 7) is intriguing, requiring further elucidation.

The current study maps the rickettsial infection process of cat fleas and establishes the baseline infection kinetics towards comparison of infection by rickettsial species and between flea species. Ultimately, *Rickettsia*- and flea-derived factors critical to infection of fleas and subsequent transmission of *R. felis* to naïve adult cat fleas can be assessed in this system.

## Competing interests

The authors declare that they have no competing interests.

## Authors’ contributions

CT performed IFA, PCR, data analysis, and wrote the manuscript. SH carried out cat flea rearing and rickettsial infected bloodmeal acquisition. VLP conducted TEM studies. LDF and KRM participated in research design, implementation, and manuscript preparation. All authors read and approved the final manuscript.
